# Gender balance in the validation of diagnostic tools for autism: A systematic review

**DOI:** 10.1192/j.eurpsy.2021.1598

**Published:** 2021-08-13

**Authors:** R. Murphy Lonergan

**Affiliations:** Edinburgh Medical School, University of Edinburgh, Edinburgh, United Kingdom

**Keywords:** autism, ASD, Gender, girls

## Abstract

**Introduction:**

Autism is a neurodevelopmental disorder that is considered more common in males; however, ascertainment estimates of ASD in the UK population suggest a significant proportion of female cases of ASD go unrecognised and undiagnosed. This review examines whether the apparent underdiagnosis of girls may be attributed to gender sampling bias in the validation of diagnostic instruments routinely used to diagnose autism.

**Objectives:**

To compare the gender ratio in validation samples of commonly used diagnostic tools for autism to estimates of the gender distribution of children with autism in the UK population

**Methods:**

A review of diagnostic tool manuals and a targeted literature search identified the gender of sample participants used to validate tools used by Scottish ASD services. Analysis of validation samples compared the mean percentage of female participants with ASD to estimates of the proportion of girls with ASD in the UK population.

**Results:**

Data on 7 tools was extracted. The mean percentage of female sample participants with ASD was significantly lower than the ascertainment estimate of females with ASD in the UK population (p=0.010, t(6)=-3.67) and significantly lower than the mean percentage of females in comparison groups (p=0.010, t(12)=-3.06).

**Conclusions:**

There is low representation of females in validity samples of tools, which may reduce their sensitivity to the female phenotype and contribute to diagnostic disparities. Future research is warranted on why instruments are poorer detectors of ASD in girls and how female features of ASD could be better represented in their structure.

